# Quality and Audience Engagement of Takotsubo Syndrome–Related Videos on TikTok: Content Analysis

**DOI:** 10.2196/39360

**Published:** 2022-09-26

**Authors:** Jing Liang, Linlin Wang, Shijie Song, Man Dong, Yidan Xu, Xinyu Zuo, Jingyi Zhang, Akil Adrian Sherif, Jafree Ehsan, Jianjun Ma, Pengyang Li

**Affiliations:** 1 Xinxiang Medical University Henan China; 2 Aerospace Central Hospital Beijing China; 3 Business School Hohai University Nanjing China; 4 The Third Affiliated Hospital of Xinxiang Medical University Henan China; 5 Divison of Cardiology, Saint Vincent Hospital Worcester, MA United States; 6 Department of Medicine Virginia Commonwealth University Richmond, VA United States; 7 Division of Cardiology, Pauley Heart Center Virginia Commonwealth University Richmond, VA United States

**Keywords:** TikTok, short video apps, information quality, Takotsubo syndrome, patient education, social media, audience engagement

## Abstract

**Background:**

The incidence of Takotsubo syndrome (TTS), also known as the broken heart syndrome or stress cardiomyopathy, is increasing worldwide. The understanding of its prognosis has been progressively evolving and currently appears to be poorer than previously thought, which has attracted the attention of researchers. An attempt to recognize the awareness of this condition among the general population drove us to analyze the dissemination of this topic on TikTok, a popular short-video–based social media platform. We found a considerable number of videos on TTS on TikTok; however, the quality of the presented information remains unknown.

**Objective:**

The aim of this study was to analyze the quality and audience engagement of TTS-related videos on TikTok.

**Methods:**

Videos on the TikTok platform were explored on August 2, 2021 to identify those related to TTS by using 6 Chinese keywords. A total of 2549 videos were found, of which 80 met our inclusion criteria and were evaluated for their characteristics, content, quality, and reliability. The quality and reliability were rated using the DISCERN instrument and the Journal of the American Medical Association (JAMA) criteria by 2 reviewers independently, and a score was assigned. Descriptive statistics were generated, and the Kruskal-Wallis test was used for statistical analysis. Multiple linear regression was performed to evaluate the association between audience engagement and other factors such as video content, video quality, and author types.

**Results:**

The scores assigned to the selected video content were low with regard to the diagnosis (0.66/2) and management (0.34/2) of TTS. The evaluated videos were found to have an average score of 36.93 out of 80 on the DISCERN instrument and 1.51 out of 4 per the JAMA criteria. None of the evaluated videos met all the JAMA criteria. The quality of the relayed information varied by source (All *P*<.05). TTS-related videos made by health care professionals accounted for 28% (22/80) of all the evaluated videos and had the highest DISCERN scores with an average of 40.59 out of 80. Multiple linear regression analysis showed that author types that identified as health professionals (exponentiated regression coefficient 17.48, 95% CI 2.29-133.52; *P*=.006) and individual science communicators (exponentiated regression coefficient 13.38, 95% CI 1.83-97.88; *P*=.01) were significant and independent determinants of audience engagement (in terms of the number of likes). Other author types of videos, video content, and DISCERN document scores were not associated with higher likes.

**Conclusions:**

We found that the quality of videos regarding TTS for patient education on TikTok is poor. Patients should be cautious about health-related information on TikTok. The formulation of a measure for video quality review is necessary, especially when the purpose of the published content is to educate and increase awareness on a health-related topic.

## Introduction

Takotsubo syndrome (TTS), also known as the broken heart syndrome or stress cardiomyopathy, is characterized by transient ventricular dysfunction with typical wall motion abnormalities [1,2]. The number of patients diagnosed with TTS has been gradually increasing [3], with hospitalizations for TTS increasing from 5.7 per 100,000 person-years in 2007 to 17.4 per 100,000 person-years in 2012 (*P*<.001) [4]. The clinical manifestation of TTS is similar to that of acute coronary syndrome (ACS) and frequently presents with chest discomfort or dyspnea, ST segment deviation on electrocardiogram, and cardiac biomarker abnormalities [2,5,6]. Given the overlap with the clinical presentation of ACS, TTS can be easily misdiagnosed. Previous reports [7,8] have shown that about 0.7%-2% of all patients with possible ACS were eventually found to have had TTS. Ongoing research has shown that the prognosis of TTS is not as benign as previously thought, with in-hospital mortality of 4.1% and long-term mortality of 24.7% [6,9].

The internet is a useful platform for effectively communicating new information, and many new technological applications have taken advantage of this to serve as a medium for patient education on health-related topics. Seeking web-based health information has become increasingly popular; many people rely on web-based resources to obtain health information and aid their medical decision-making [10]. Research has shown that health outcomes can be positively impacted by appropriately and effectively utilizing social media platforms [11]. One of the many social media applications that has been used to reach a large audience is TikTok [12]. However, the most important limitation of such platforms is the unreliable quality of the information presented. Anyone can present information on social media platforms, most of which lack formal moderation for authenticity and reliability of the presented material [13]. Recent systematic reviews have suggested that the quality of web-based health information is problematic and perhaps made worse when considering information disseminated on social media platforms [14].

Along with the quality of information, audience engagement is another key component of effective web-based health communication. Audience engagement in health-related topics has been studied in traditional social media platforms such as YouTube and Facebook [15-19]. The influence of many factors such as video content, quality, and information sources on audience engagement has been studied previously [15,16,18-20]. Several studies have shown that video content is associated with audience engagement [15,18,20]. Szmuda et al [15] studied the association between video content and audience engagement in COVID-19 videos on YouTube, and they found that videos showing the causes, management strategies, diagrams, and structure anatomies were associated with a higher “like” ratio. Another study [20] on audience engagement and COVID-19 short videos on TikTok found that content type (news, codebook, etc) influenced the level of audience engagement.

Previous studies have yielded inconsistent conclusions on the impact of video quality on audience engagement. A study on videos of stroke on YouTube by Szmuda et al [16] showed that there was no strong correlation between the DISCERN score (an indicator of video quality) and audience engagement. Huang et al [19] found that nephrolithiasis-related videos on YouTube with inaccurate statements were associated with higher audience engagement (viewer-generated comments, thumbs-up and thumbs-down ratings) than videos without inaccuracies. The source of information was also found to affect audience engagement. Szmuda et al [16] found that higher engagement was noted in stroke-related videos that were uploaded by an educational channel on YouTube. Recently, studies [21,22] have shown that the emerging short-video social apps can satisfy people’s intrinsic motivations and elicit user engagement when disseminating health information.

TikTok (DouYin in Chinese) is a short-video social app with a sizable userbase wherein individual users create and publicly post short videos on various subjects. Initially, when the platform first became popular, the video length was limited to short 60-second clips. However, with growing demand, the length was extended to allow up to 5-minute-long videoclips [23]. TikTok has attained significant popularity since its launch in September 2016 and has since raked up more than 500 million active users and 1 billion downloads [24,25]. Given its extensive reach and better audience engagement than other traditional social media platforms [23], TikTok has the potential to be a great source of health information dissemination and become increasingly popular among general health consumers as an emerging health information source. Several studies evaluating patient education videos regarding COVID-19 [26], skin-related diseases [27], chronic obstructive pulmonary disease [28], and aesthetic surgery procedures [29] posted on TikTok have been published. These studies have revealed that the overall quality of such videos presenting health information on TikTok is low, and some even present overtly false information [27,29]. Very few studies [15,16,18,20] have focused on audience engagement with health care information on TikTok as compared to that with health care information on other social media platforms. We found a considerable number of videos regarding TTS on TikTok; however, the quality and content of the presented information and whether these factors may affect audience engagement remain largely unknown. Therefore, this study aims to evaluate the content and quality of videos related to TTS on TikTok and assess the qualitative metrics that drive audience engagement (in terms of the number of likes) with a video.

## Methods

### Search Strategy

**Figure 1:**
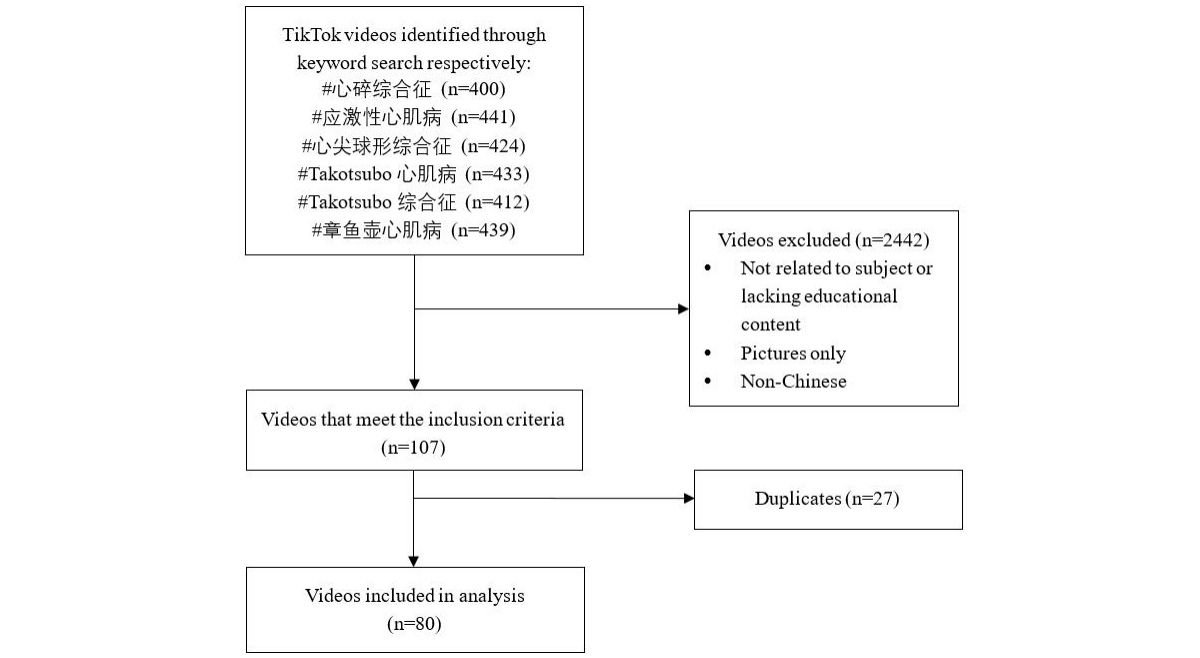
Selection process implemented in this study.

The search was conducted on August 2, 2021, in China. Six specific hashtags（“#应激性心肌病” “#Takotsubo综合征” “#Takotsubo心肌病” “#心碎综合征” “#章鱼壶心肌病” “#心尖球形综合征”）that refer to TTS in Chinese were used to retrieve TikTok videos related to TTS. TikTok provides 3 ways to filter videos, that is, overall ranking, most recent, and most liked. Considering that most users use the default sorting option, “overall ranking,” we performed the search in TikTok under the discover mode using the “overall ranking” sort option. All the resultant videos for each keyword were retrieved and screened. The initial search returned 2549 videos (441, 400, 433, 412, 439, and 424, respectively), of which 80 videos met our criteria for analysis after screening. The exclusion criteria were as follows: (1) videos not related to TTS or lacking educational information, (2) duplicate videos, (3) videos not in Chinese, and (4) videos that were not original. Figure 1 illustrates the selection process implemented in our study.

### Data Extraction

Baseline characteristics were extracted directly from each video and the video author’s public TikTok profile on the same day of the data search. With respect to the video authors, we collected their account ID, inauguration unit, number of both followers and those they follow, overall number of posted videos, and likes obtained. Additionally, we further ascertained whether the public TikTok profile had photos, live broadcast information, and contact details. For each individual video, we also collected the uniform resource locator, the date posted, the number of likes, comments, reposts, and duration. Based on the abovementioned information and historical videos on their profile page, the TikTok authors were classified into 6 categories: (1) individual science communicators, (2) news agencies, (3) for-profit organizations, (4) health professionals, (5) nonprofit organizations, and, (6) general users. Furthermore, the content of each video was assessed for the following characteristics: use of characters, background music, emoji, flash, and video subtitles.

### Coding Schema

We used the DISCERN instrument and Journal of the American Medical Association (JAMA) criteria to evaluate the quality of the selected TikTok videos. These instruments are commonly used standards for health information evaluation [30,31]. The DISCERN instrument (quality criteria for consumer health information) consists of 16 questions, with each question scored from 1 to 5 points. Questions are divided into 3 parts: reliability of the publication (questions 1 to 8), quality of information about treatment options (questions 9 to 15), and the overall score of the publication (question 16) [32]. The JAMA benchmarks were applied to evaluate health-related information’s reliability, plausibility, and usefulness in the internet [33]. The JAMA criteria consist of 4 main sections, which are scored from 0 to 4. Six questions obtained from reports of Goobie et al [34] were used to measure the quality of the video content. The 6 questions assessed the definition, signs or symptoms, risk factors, evaluation, management, and outcomes of the disease discussed in the videos. Each aspect was scored across 3 degrees from 0 to 2: not addressed, partially addressed, and sufficiently addressed.

### Video Coding

All video contents evaluated using the DISCERN instrument, JAMA benchmarks, and the 6 questions from the studies of Goobie et al [34] were independently scored and coded by 2 study authors (JL and YX). Prior to coding, a training exercise was conducted, during which 20 videos each were scored and coded independently by the 2 coding authors, and any resulting discrepancies were addressed and resolved to ensure homogeneity in coding. An average of 2 points was assigned by rounding to the nearest unit. Microsoft Excel (2019) was used to extract, code the basic information of each video, and process statistical data.

### Statistical Analysis

Descriptive statistics for continuous variables, including the mean, median, range, and standard deviation, were calculated. SPSS version 22.0 (IBM Corp) was used to perform data analysis. An intraclass correlation coefficient was used to assess the interrater agreement. The average agreement by intraclass correlation coefficient was 0.87 for content rating, 0.92 for DISCERN scales rating, and 0.88 for the JAMA benchmark rating. The average intraclass correlation coefficients for interrater agreements all exceeded the recommended value of 0.75, indicating that the ratings had good reliability [35]. The Kruskal-Wallis test was performed to identify differences between the extracted variables. A *P* value <.05 was deemed statistically significant. In our paper, we use the number of likes as a proxy measure of audience engagement. Multiple linear regression was performed to evaluate the association between audience engagement and other factors such as video quality and author types. The regression analysis was conducted on R (R version 4.2.1; 2022-06-23 ucrt), with a log transformation of the outcome variable audience engagement. This study did not involve human subjects, and hence, the study was not reviewed by the institutional review board.

## Results

### Video Characteristics

The average length of the evaluated videos was 59 seconds, with a maximum duration of 700 seconds and a minimum duration of 7 seconds. On average, a video received 5543 likes and 635 reposts. The majority of the videos (49/80, 61%) had the presence of people, 90% (72/80) had subtitles, and 70% (56/80) of the videos had background music. Approximately 19% (15/80) and 23% (18/80) of the videos had emojis and animations, respectively (Table 1).

With regard to content creation, users classified as health professionals posted the most videos (22/80, 28%), followed by general users (20/80, 25%), individual science communicators (18/80, 23%), news agencies (11/80, 14%), and for-profit organizations (6/80, 8%). Nonprofit organizations posted the fewest videos (3/80, 4%) (Multimedia Appendix 1). The average number of likes received per video, categorized by video author types, from high to low were as follows: news agencies, health professionals, for-profit organizations, individual science communicators, general users, and nonprofit organizations (Table 2).

**Table 1:** Basic characteristics of the Takotsubo syndrome–related videos on TikTok.

		Mean	Median
**Author’s account information**			
	Likes	3,451,837	188,000
	Following	166	48
	Followers	622,697	41,000
	Videos	743	141
**Characteristics of Takotsubo syndrome–related videos**			
	Duration (min)	59	41
	Likes	5543	108
	Comments	518	8
	Reposts	635	8
**Information quality**			
	Six questions	5.40	N/A^a^
	DISCERN score	36.93	N/A
	Journal of the American Medical Association criteria	1.51	N/A

^a^N/A: not applicable.

**Table 2:** Characteristics of Takotsubo syndrome–related videos on TikTok categorized by source.

		Health professionals (n=22)		Individual science communicators (n=18)		General users (n=20)		News agencies (n=11)		For-profit organizations (n=6)		Nonprofit organizations (n=3)
**Author’s account information (mean)**												
	Likes	1,764,734	2,029,657		115,554		17,184,623		4,066,700		61,000	
	Following	168	64		343		104		114		19	
	Followers	1,513,977	192,388		26,012		1,022,507		288,864		12,831	
	Videos	319	101		97		3969		1028		740	
**Characteristics of Takotsubo syndrome–related videos (mean)**												
	Duration (min)	47	46		89		57		46		45	
	Likes	10,048	3326		163		11,861		4761		82	
	Comments	1066	229		10		1002		213		39	
	Reposts	1473	294		8		1113		243		43	
**Video production, n (%)**												
	Presence of people	20 (91)	7 (39)		12 (60)		9 (82)		0 (0)		1 (33)	
	Background music	12 (55)	15 (83)		15 (75)		8 (73)		4 (67)		2 (67)	
	On-screen emoji	6 (27)	4 (22)		2 (10)		0 (0)		3 (50)		0 (0)	
	Animation	1 (5)	8 (44)		5 (25)		0 (0)		4 (67)		0 (0)	
	Subtitles	21 (96)	18 (100)		17 (85)		9 (82)		5 (83)		2 (67)	

### Video Content

The average total score of Goobie et al’s [34] 6-question survey by both raters was 5.40 out of 12, indicating that the overall content quality of these videos was average (in our study, the average total score of Goobie et al’s [34] 6-question survey ≥9.0 is considered excellent, ≥6.0-<9.0 is considered good, and <6.0 is considered average). The average scores for the videos given by the 2 raters for each of the 6 criteria described by Goobie et al [34] are shown in Multimedia Appendix 2. Of the 6 items, “symptoms” was the most common, while “diagnosis” was the least common. The average scores (total score for each item is 2), graded for each item and ranked from highest to lowest were as follows: symptoms (1.42), risk factors (1.09), definition (1.06), outcome (0.83), management (0.66), and diagnosis (0.34). We further analyzed video authors by categorizing them into the professional and nonprofessional group. Health professionals comprised the professional group, while the nonprofessional group included authors other than health professionals, such as news agencies and for-profit organizations. Health professionals scored higher in terms of video content compared to the other 5 author categories (Table 3). Videos posted by professionals (6.20) scored higher on average than those posted by nonprofessionals (5.09).

**Table 3:** Scoring on various instruments for videos related to Takotsubo syndrome categorized by sources.

	Health professionals	Individual science communicators	General users	News agencies	For-profit organizations	Nonprofit organizations	*P* value^a^
Six questions	6.20	4.81	4.65	5.77	6.08	5.33	<.001
DISCERN score	40.59	36.78	30.80	38.36	40.33	39.67	<.001
JAMA^b^ criteria	1.91	1.08	1.08	1.77	1.83	2.00	<.001

^a^*P* value obtained by performing the Kruskal-Wallis test; null hypothesis: no difference among the average scores for the 6 groups. It assumes that the 2 coders performed a homogeneous assessment

^b^JAMA: Journal of the American Medical Association.

### Video Quality

The average score given for the videos on the DISCERN instrument by both raters was 36.93 out of 80, indicating that the overall quality of these videos was poor (average total score of 16-26 is very poor, a score of 27-38 is poor, a score of 39-50 is fair, a score of 51-62 is good, and a score >63 is excellent [36]). Among videos created by different author types, the average score given to videos made by health professionals was the highest (40.59), followed by for-profit organizations (40.33) and non-profit organizations (39.67). Although the video scores of the above 3 resources are relatively higher, the quality rating level is only fair. The lowest average score was for those created by general users (30.80), indicating that videos posted by general users were the poorest in terms of quality. The comparison of scores between the professional and nonprofessional group is shown in Multimedia Appendix 3. Questions 3, 6, and 8 were scored above 3 points, and these questions addressed relevance, bias, and areas of uncertainty with reference to the selected videos, respectively. Questions 5 and 9-14 were found to have scores of less than 2 points. These questions mainly addressed whether the date of the information used or reported in the publication was clear; whether the mechanism, benefits, and risks of each treatment, consequences of not treating, or impact of different treatment options on the overall quality of life were described; and whether the possibility of more than one treatment choice being available was clear (Multimedia Appendix 3).

Both raters agreed that 98% (78/80) of the videos provided the latest information, 43% (34/80) of the videos provided information regarding authorship, but none mentioned the disclosure statement. None of the videos met all the JAMA criteria. The average JAMA score was 1.51. By video author categories, an average score of 1.91 was obtained by health professionals, 1.08 by individual science communicators, 1.08 by general users, 1.77 by news agencies, 1.83 by for-profit organizations, and 2.00 by nonprofit organizations. Based on the Kruskal-Wallis test, there were statistically significant differences in content, DISCERN instrument scores, and the JAMA standard video assessment scores among the 6 author groups (*P*<.05).

### Analysis of Information Quality and Audience Engagement

The results of our multiple linear regression analysis showed that author types that identified as health professionals (exponentiated regression coefficient 17.48, 95% CI 2.29-133.52; *P*=.006) and individual science communicators (exponentiated regression coefficient 13.38, 95% CI 1.83-97.88; *P*=.01) were significant and independent determinants of audience engagement (in terms of the number of likes). Other author types of videos, video content, and DISCERN document scores were not associated with higher likes (Table 4).

**Table 4:** Multiple linear regression analysis on audience engagement^a^.

Variable		Log-transformed data on audience engagement				Original audience engagement		*P* value
		Estimated intercept	SE	*t* (*df*)	95% CI	Estimated intercept	95% CI	
**Author types**								
	Intercept	4.1848	2.5466	1.643 (70)	–0.89 to 9.26	65.68	0.41 to 10550.22	.11
	Health professionals	2.8609	1.0195	2.806 (70)	0.83 to 4.89	17.48	2.29 to 133.52	.006
	Individual science communicators	2.5937	0.9978	2.599 (70)	0.60 to 4.58	13.38	1.83 to 97.88	.01
	News agencies	0.638	1.0957	0.582 (70)	–1.55 to 2.82	1.89	0.21 to 16.83	.56
	For-profit organizations	1.8838	1.3658	1.379 (70)	–0.84 to 4.61	6.58	0.43 to 100.26	.17
	Nonprofit organizations	0.8744	1.8097	0.483 (70)	–2.73 to 4.48	2.40	0.06 to 88.56	.63
	Video content^b^	0.4602	0.3158	1.457 (70)	–0.17 to 1.09	1.58	0.84 to 2.97	.15
**DISCERN instrument**								
	Reliability of the videos (items 1-8)	–0.6326	1.3684	–0.462 (70)	–3.36 to 2.10	0.53	0.03 to 8.14	.65
	Quality of treatment choices (items 9-15)	–1.1136	1.3283	–0.838 (70)	–3.76 to 1.54	0.33	0.02 to 4.64	.41
	Overall information quality (item 16)	0.3339	0.8090	0.413 (70)	–1.28 to 1.95	1.40	0.28 to 7.01	.68

^a^Residual standard error: 2.632 on 70 degrees of freedom; multiple *R*^2^=0.2048; adjusted *R*^2^=0.1025; *F*_9,70_=2.003; *P*=.05.

^b^ Six questions obtained from reports of Goobie et al [34] were used to measure the video content.

## Discussion

### Principal Findings

Growing evidence suggests that TTS is a severe cardiac disorder with a substantial mortality risk. A meta-analysis showed no difference in the in-hospital and long-term mortality between TTS and ACS [37]. Long-term recurrence rates of TTS have been reported to be as high as 11.4% over 4 years [38]. Although regional wall dysfunction is reversible in TTS, patients may continue to experience chest pain, fatigue, and dyspnea even after recovery of wall function [39]. Notably, traditional cardiovascular risk factors are less commonly seen in TTS compared with those seen in ACS [37]. TTS presentations are often instigated by stress-related emotional and physical factors preceding symptoms [1]. Although such factors may not always be preventable, awareness of TTS as an entity by the general population may improve the measures adopted by individuals in its recognition and seeking prompt medical attention. Additionally, it may improve the collateral history provided by affected individuals, which may aid health care providers in its diagnosis. This is especially important as patients who develop TTS are known to be more apathetic with regard to their mortality and their acute presentation, as evidenced by a study [40] evaluating the psychology of patients with TTS and ACS. That study [40] has also shown that patients with TTS were less likely to be concerned about contracting diseases (*P*<.05) and had fewer thoughts related to the acute cardiac episode that interfered with their life (*P*<.001). It is still unclear whether awareness of TTS will change these perceptions, but an increase in awareness may certainly encourage individuals to adopt measures to mitigate individual stressors, which, in turn, may reduce the incidence of TTS. Some patients with TTS continue to experience symptoms despite resolution of the acute phase. Cardiac rehabilitation may be beneficial for these patients to improve the quality of life and reduce episodes of ongoing chest pain, but only few patients have been reported to receive cardiac rehabilitation owing to the lack of awareness among patients and even doctors [41]. Improving the awareness of TTS may aid the rate of those seeking cardiac rehabilitation for persistent symptoms as well. There is an increasing trend toward using social media for patient education. Despite there being a lot of relevant information on social media platforms from reliable sources, there also exists a large amount of inaccurate information. This poses a significant challenge to users seeking easily accessible and comprehensible information regarding their health. Moreover, low-quality information weakens the ability of people to make informed decisions and can even lead to harmful consequences.

### Video Quality Analysis

To the best of our knowledge, this is the first study to analyze the quality of TTS-related videos on TikTok. As a popular social media platform, TikTok has the potential to become a valuable and influential platform to disseminate health information, especially in the context of the current COVID-19 pandemic [42,43]. In our study, 80 TTS-related videos received 443,469 likes and were commented on and shared thousands of times, which also affirms TikTok’s powerful communication capabilities. However, there is significant concern regarding the quality of these TTS videos on TikTok. Their scores on the DISCERN instrument (36.93) and the JAMA criteria (1.51) were generally low. These findings are similar to the results of Śledzińska et al [44] in their study on YouTube videos (n=61) on meningioma treatment. In their study, the mean total DISCERN score was poor as well at 36.4. Part of the reason for the poor quality may be that short-video platforms lack scientific insight for health information dissemination. Furthermore, there is no restriction on the type of content that video authors may publish nor is there any restriction based on author type to ensure content quality. Addressing these concerns in the context of health information dissemination will be invaluable [43]. Considering this, we further studied the quality of videos based on author type. We found that health professionals are the leading creators of popular science videos (Multimedia Appendix 1). Videos created by health professionals had higher average DISCERN scores than those created by non–health professionals. This is consistent with current literature reports that videos created by professionals are likely to have higher quality [29,45,46]. Given the inconsistency in the quality of videos based on the source, we recommend that patients be cautious when obtaining health-related information through platforms such as TikTok.

Notably, videos created by health professionals also received the highest number of likes and reposts in our study, which is in contrast to the results of other prior studies. In previous studies evaluating videos on psoriasis and nephrolithiasis on YouTube, poor-quality videos received greater attention [19,47]. Our study shows that higher health professional participation in health-related short video productions results in higher popularization ranks, having gained wider user attention. Although the quality of the videos produced by health professionals needs to be improved, it is undeniable that the participation of medical professionals in the creation of high-quality videos plays an essential role in promoting health education [29].

### Content and Optimization Analysis

In terms of video content, we found that very few videos introduced the concept of diagnosis of TTS, and the display of reference information sources was not common, which remains an area to be addressed. The comprehensiveness and accuracy of the video content is a necessary prerequisite to ensure reliable transmission of information, especially when most TikTok users do not have the ability to differentiate health information for reliability. Therefore, we recommend that video producers provide sources of reference information. In addition, in the process of screening videos, we noticed that many videos showed the causes of TTS to be mainly emotional factors, and only a few videos emphasized physical factors, which are important and cannot be ignored. A retrospective study on patients with TTS showed that physical factors are considerable risk factors for in-patient mortality [48]. It is appropriate for video producers to ask experts for review before content creation or check authoritative source materials to ensure that the information published is comprehensive and accurate. As put forward by Oh and You [49], it may be essential to form an expert evaluation team to authenticate the reliability of health-related videos prior to dissemination and provide corresponding identification certificates. In addition, organizational formats of health information can affect the health intervention. Therefore, video authors should fully understand individuals’ needs regarding health information and organize information effectively to provide targeted health information [50]. The video monitoring platform should also design a recommendation algorithm that filters higher-quality videos for users as much as possible, especially with reference to medical- and health-related information.

### Audience Engagement Analysis

Through multiple linear regression analysis, we found that, compared with videos made by general authors, those made by health professionals and individual science communicators were more likely to obtain likes. Interestingly, no correlation was found between audience engagement and a video’s DISCERN scores. The number of likes is commonly viewed as a collective filter and as an indicator of popularity, which may reflect video quality [19,51,52]. However, our findings suggested that, similar to that in other platforms, the audience on the TikTok platform pay more attention to the identity of the author rather than the content and quality of the video. This finding echoes with those of previous studies [16,19], which show that consumers should remain cautious of using such indictors to judge a video’s credibility and that health professionals and individual science communicators have a significant role in video production in TikTok. By providing high-quality videos through these professional authors, TikTok can allow for accelerated health care information dissemination and even potentially improve outcomes in certain diseases.

### Limitations and Future Directions

Our study has some limitations. First, this was a cross-sectional study with a small sample size, despite attempts to include all relevant videos. Second, we only evaluated Chinese videos, which may not be representative in a global context. Future research studies can target characteristics of videos in various languages and regions. In addition, we utilized common standards that are currently used for health information video evaluation, namely, the DISCERN instrument and the JAMA criteria [30,34]. However, some scholars have suggested that these 2 standards were developed relatively early and were initially used to evaluate website information and may have limitations when used to evaluate video information [53]. It is vital that a new video information evaluation tool is developed to adapt in this era of rapidly proliferating video content. Finally, we did not analyze the user comments addressed in each video, and we were unable to track the behavioral and psychological changes of the end user or the information recipient. How these videos affect patient behavior in reality is an area that needs to be studied in the future.

### Conclusion

By analyzing the quality of TTS-related videos on TikTok, we found that videos produced by health professionals were found to have the highest DISCERN scores. However, the overall quality of the videos related to TTS on TikTok is poor. The multiple linear regression analysis showed that author type categories of health professionals (*P*=.006) and individual science communicators (*P*=.01) were significant and independent determinants of consumer engagement (in terms of the number of likes). Our study indicates that the formulation of a measure to review video quality and reliability, especially with respect to health-related information dissemination on TikTok platform, is imperative and patients should be cautious when obtaining health-related information through TikTok. Medical professionals and individual science communicators should be encouraged to create high-quality health-related videos, which may potentially have higher audience engagement and promote health education.

## Appendix

Multimedia Appendix 1 Video authorship of Takotsubo syndrome–related videos.

Multimedia Appendix 2 Takotsubo syndrome–related videos on TikTok scored per criteria described by Goobie et al [34].

Multimedia Appendix 3 The average DISCERN score per question categorized by groups.

## Abbreviations

ACS: acute coronary syndrome
JAMA: Journal of the American Medical Association
TTS: Takotsubo syndrome

## References

[1] Ghadri JR, Cammann VL, Jurisic S, Seifert B, Napp LC, Diekmann J, Bataiosu DR, D'Ascenzo F, Ding KJ, Sarcon A, Kazemian E, Birri T, Ruschitzka F, Lüscher Thomas F, Templin C, InterTAK co-investigators (2017). A novel clinical score (InterTAK Diagnostic Score) to differentiate takotsubo syndrome from acute coronary syndrome: results from the International Takotsubo Registry. Eur J Heart Fail.

[2] Ghadri J, Wittstein IS, Prasad A, Sharkey S, Dote K, Akashi YJ, Cammann VL, Crea F, Galiuto L, Desmet W, Yoshida T, Manfredini R, Eitel I, Kosuge M, Nef HM, Deshmukh A, Lerman A, Bossone E, Citro R, Ueyama T, Corrado D, Kurisu S, Ruschitzka F, Winchester D, Lyon AR, Omerovic E, Bax JJ, Meimoun P, Tarantini G, Rihal C, Y-Hassan S, Migliore F, Horowitz JD, Shimokawa H, Lüscher Thomas Felix, Templin C (2018). International Expert Consensus Document on Takotsubo Syndrome (Part I): Clinical Characteristics, Diagnostic Criteria, and Pathophysiology. Eur Heart J.

[3] Shao Yangzhen, Redfors Bjorn, Lyon Alexander R, Rosengren Annika, Swedberg Karl, Omerovic Elmir (2012). Trends in publications on stress-induced cardiomyopathy. Int J Cardiol.

[4] Murugiah K, Wang Y, Desai NR, Spatz ES, Nuti SV, Dreyer RP, Krumholz HM (2016). Trends in Short- and Long-Term Outcomes for Takotsubo Cardiomyopathy Among Medicare Fee-for-Service Beneficiaries, 2007 to 2012. JACC Heart Fail.

[5] Prasad A, Lerman A, Rihal CS (2008). Apical ballooning syndrome (Tako-Tsubo or stress cardiomyopathy): a mimic of acute myocardial infarction. Am Heart J.

[6] Templin C, Ghadri JR, Diekmann J, Napp LC, Bataiosu DR, Jaguszewski M, Cammann VL, Sarcon A, Geyer V, Neumann CA, Seifert B, Hellermann J, Schwyzer M, Eisenhardt K, Jenewein J, Franke J, Katus HA, Burgdorf C, Schunkert H, Moeller C, Thiele H, Bauersachs J, Tschöpe Carsten, Schultheiss H, Laney CA, Rajan L, Michels G, Pfister R, Ukena C, Böhm Michael, Erbel R, Cuneo A, Kuck K, Jacobshagen C, Hasenfuss G, Karakas M, Koenig W, Rottbauer W, Said SM, Braun-Dullaeus RC, Cuculi F, Banning A, Fischer TA, Vasankari T, Airaksinen KEJ, Fijalkowski M, Rynkiewicz A, Pawlak M, Opolski G, Dworakowski R, MacCarthy P, Kaiser C, Osswald S, Galiuto L, Crea F, Dichtl W, Franz WM, Empen K, Felix SB, Delmas C, Lairez O, Erne P, Bax JJ, Ford I, Ruschitzka F, Prasad A, Lüscher Thomas F (2015). Clinical Features and Outcomes of Takotsubo (Stress) Cardiomyopathy. N Engl J Med.

[7] Pillière Rémy, Mansencal N, Digne F, Lacombe P, Joseph T, Dubourg O (2006). Prevalence of tako-tsubo syndrome in a large urban agglomeration. Am J Cardiol.

[8] Azzarelli S, Galassi AR, Amico F, Giacoppo M, Argentino V, Tomasello SD, Tamburino C, Fiscella A (2006). Clinical features of transient left ventricular apical ballooning. Am J Cardiol.

[9] Stiermaier T, Moeller C, Oehler K, Desch S, Graf T, Eitel C, Vonthein R, Schuler G, Thiele H, Eitel I (2016). Long-term excess mortality in takotsubo cardiomyopathy: predictors, causes and clinical consequences. Eur J Heart Fail.

[10] Jiang S, Liu J (2020). Examining the relationship between Internet health information seeking and patient-centered communication in China: taking into account self-efficacy in medical decision-making. Chinese Journal of Communication.

[11] Merolli M, Gray K, Martin-Sanchez F (2013). Health outcomes and related effects of using social media in chronic disease management: a literature review and analysis of affordances. J Biomed Inform.

[12] Kong W, Song S, Zhao YC, Zhu Q, Sha L (2021). TikTok as a Health Information Source: Assessment of the Quality of Information in Diabetes-Related Videos. J Med Internet Res.

[13] Ouyang Y (2016). Student's death highlights gaps in China's health regulations. The Lancet Oncology.

[14] Zhang Y, Sun Y, Xie B (2015). Quality of health information for consumers on the web: A systematic review of indicators, criteria, tools, and evaluation results. J Assn Inf Sci Tec.

[15] Szmuda T, Syed MT, Singh A, Ali S, Özdemir Cathrine, Słoniewski P (2020). YouTube as a source of patient information for Coronavirus Disease (COVID-19): A content-quality and audience engagement analysis. Rev Med Virol.

[16] Szmuda T, Alkhater A, Albrahim M, Alquraya E, Ali S, Dunquwah RA, Słoniewski P (2020). YouTube as a source of patient information for stroke: A content-quality and an audience engagement analysis. J Stroke Cerebrovasc Dis.

[17] Sedgewick JA, Arnold EP, Stamm MA, Mulcahey MK (2022). Orthopaedic Sports Medicine Podcasts Should Tailor Characteristics Such as Episode Length and Social Media Utilization for Best Audience Engagement. Arthrosc Sports Med Rehabil.

[18] Szmuda T, Özdemir Cathrine, Fedorow K, Ali S, Słoniewski P (2021). YouTube as a source of information for narcolepsy: A content-quality and optimization analysis. J Sleep Res.

[19] Huang MM, Winoker JS, Allaf ME, Matlaga BR, Koo K (2021). Evidence-based quality and accuracy of YouTube videos about nephrolithiasis. BJU Int.

[20] Chen Q, Min C, Zhang W, Ma X, Evans R (2021). Factors Driving Citizen Engagement With Government TikTok Accounts During the COVID-19 Pandemic: Model Development and Analysis. J Med Internet Res.

[21] Song S, Zhao YC, Yao X, Ba Z, Zhu Q (2021). Short video apps as a health information source: an investigation of affordances, user experience and users’ intention to continue the use of TikTok. INTR.

[22] Song S, Zhao YC, Yao X, Ba Z, Zhu Q (2021). Serious information in hedonic social applications: affordances, self-determination and health information adoption in TikTok. JD.

[23] Stokel-Walker C TikTok wants longer videos-whether you like it or not. Wired.com.

[24] Lovett JT, Munawar K, Mohammed S, Prabhu V (2021). Radiology Content on TikTok: Current Use of a Novel Video-Based Social Media Platform and Opportunities for Radiology. Curr Probl Diagn Radiol.

[25] Zheng DX, Mulligan KM, Scott JF (2021). TikTok and dermatology: An opportunity for public health engagement. J Am Acad Dermatol.

[26] Basch CH, Mohlman J, Fera J, Tang H, Pellicane A, Basch CE (2021). Community Mitigation of COVID-19 and Portrayal of Testing on TikTok: Descriptive Study. JMIR Public Health Surveill.

[27] Chen MKY, Garden F, Sebaratnam DF (2021). Isotretinoin on TikTok™: misinformation getting under our skin. Clin Exp Dermatol.

[28] Song S, Xue X, Zhao YC, Li J, Zhu Q, Zhao M (2021). Short-Video Apps as a Health Information Source for Chronic Obstructive Pulmonary Disease: Information Quality Assessment of TikTok Videos. J Med Internet Res.

[29] Om A, Ijeoma B, Kebede S, Losken A (2021). Analyzing the Quality of Aesthetic Surgery Procedure Videos on TikTok. Aesthet Surg J.

[30] Eksi Ozsoy H (2021). Evaluation of YouTube videos about smile design using the DISCERN tool and Journal of the American Medical Association benchmarks. J Prosthet Dent.

[31] Stern J, Georgsson S, Carlsson T (2021). Quality of web-based information at the beginning of a global pandemic: a cross-sectional infodemiology study investigating preventive measures and self care methods of the coronavirus disease 2019. BMC Public Health.

[32] Charnock D, Shepperd S, Needham G, Gann R (1999). DISCERN: an instrument for judging the quality of written consumer health information on treatment choices. J Epidemiol Community Health.

[33] Silberg WM, Lundberg GD, Musacchio RA (1997). Assessing, controlling, and assuring the quality of medical information on the Internet: Caveant lector et viewor--Let the reader and viewer beware. JAMA.

[34] Goobie GC, Guler SA, Johannson KA, Fisher JH, Ryerson CJ (2019). YouTube Videos as a Source of Misinformation on Idiopathic Pulmonary Fibrosis. Ann Am Thorac Soc.

[35] Koo TK, Li MY (2016). A Guideline of Selecting and Reporting Intraclass Correlation Coefficients for Reliability Research. J Chiropr Med.

[36] Olkun HK, Ari Demirkaya A (2018). Evaluation of Internet Information about Lingual Orthodontics Using DISCERN and JAMA Tools. Turk J Orthod.

[37] Han P, Yang Z, Diao K, Huang S, Shen M, Zhang Y, He S, Guo Y (2020). Comparison of clinical profiles between takotsubo syndrome and acute coronary syndrome: a systematic review and meta-analysis. Heart Fail Rev.

[38] Elesber AA, Prasad A, Lennon RJ, Wright RS, Lerman A, Rihal CS (2007). Four-year recurrence rate and prognosis of the apical ballooning syndrome. J Am Coll Cardiol.

[39] Singh K (2016). Tako-Tsubo syndrome: issue of incomplete recovery and recurrence. Eur J Heart Fail.

[40] Gorini A, Galli F, Giuliani M, Pierobon A, Werba JP, Adriano EP, Trabattoni D (2022). Psychological Characteristics of Patients with Takotsubo Syndrome and Patients with Acute Coronary Syndrome: An Explorative Study toward a Better Personalized Care. J Pers Med.

[41] Wu CM, McKeon J, Abbott JD, Jiang L, Wu W (2019). Referral to Cardiac Rehabilitation and Outcomes for Patients With Takotsubo Cardiomyopathy. J Cardiopulm Rehabil Prev.

[42] Basch CH, Hillyer GC, Jaime C (2020). COVID-19 on TikTok: harnessing an emerging social media platform to convey important public health messages. Int J Adolesc Med Health.

[43] Eghtesadi M, Florea A (2020). Facebook, Instagram, Reddit and TikTok: a proposal for health authorities to integrate popular social media platforms in contingency planning amid a global pandemic outbreak. Can J Public Health.

[44] Śledzińska P, Bebyn MG, Furtak J (2021). Quality of YouTube Videos on Meningioma Treatment Using the DISCERN Instrument. World Neurosurg.

[45] Villa-Ruiz C, Kassamali B, Mazori DR, Min M, Cobos G, LaChance A (2021). Overview of TikTok's most viewed dermatologic content and assessment of its reliability. J Am Acad Dermatol.

[46] Stellefson M, Chaney B, Ochipa K, Chaney D, Haider Z, Hanik B, Chavarria E, Bernhardt JM (2014). YouTube as a source of chronic obstructive pulmonary disease patient education: a social media content analysis. Chron Respir Dis.

[47] Mueller SM, Jungo P, Cajacob L, Schwegler S, Itin P, Brandt O (2019). The Absence of Evidence is Evidence of Non-Sense: Cross-Sectional Study on the Quality of Psoriasis-Related Videos on YouTube and Their Reception by Health Seekers. J Med Internet Res.

[48] Sobue Y, Watanabe E, Ichikawa T, Koshikawa M, Yamamoto M, Harada M, Ozaki Y (2017). Physically triggered Takotsubo cardiomyopathy has a higher in-hospital mortality rate. Int J Cardiol.

[49] Oh J, You SY (2021). Febrile seizure: What information can caregivers access through YouTube?. Seizure.

[50] Xu X, Yang M, Zhao YC, Zhu Q (2020). Effects of message framing and evidence type on health information behavior: the case of promoting HPV vaccination. AJIM.

[51] Yang S, Brossard D, Scheufele DA, Xenos MA (2022). The science of YouTube: What factors influence user engagement with online science videos?. PLoS One.

[52] Erdem MN, Karaca S (2018). Evaluating the Accuracy and Quality of the Information in Kyphosis Videos Shared on YouTube. Spine (Phila Pa 1976).

[53] Azer SA (2020). Are DISCERN and JAMA Suitable Instruments for Assessing YouTube Videos on Thyroid Cancer? Methodological Concerns. J Cancer Educ.

