# Supplementary figures and images for: Quality and Audience Engagement of Takotsubo Syndrome–Related Videos on TikTok: Content Analysis
Source: J Med Internet Res. 2022 Sep 26;24(9):e39360. doi: 10.2196/39360 (PMC9555329; doi:10.2196/39360)

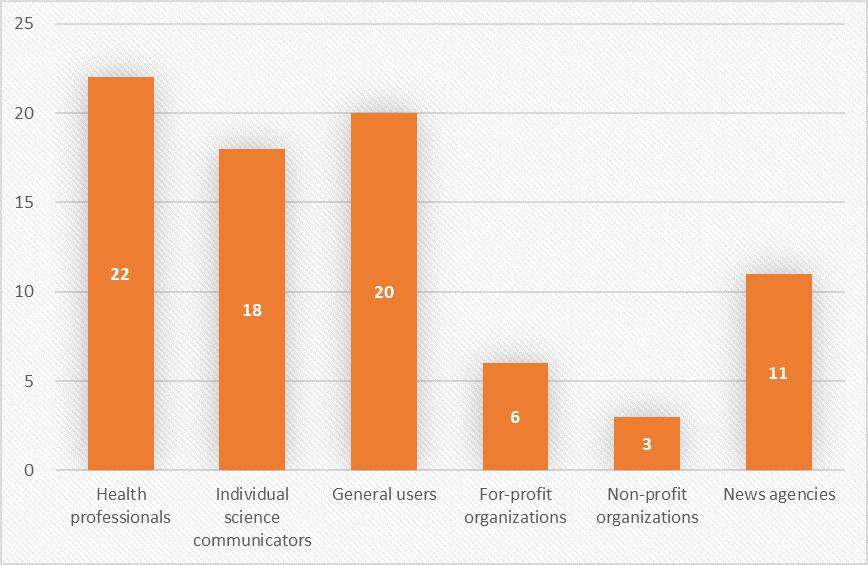

Supplement: Multimedia Appendix 1 [file jmir_v24i9e39360_app1.png]

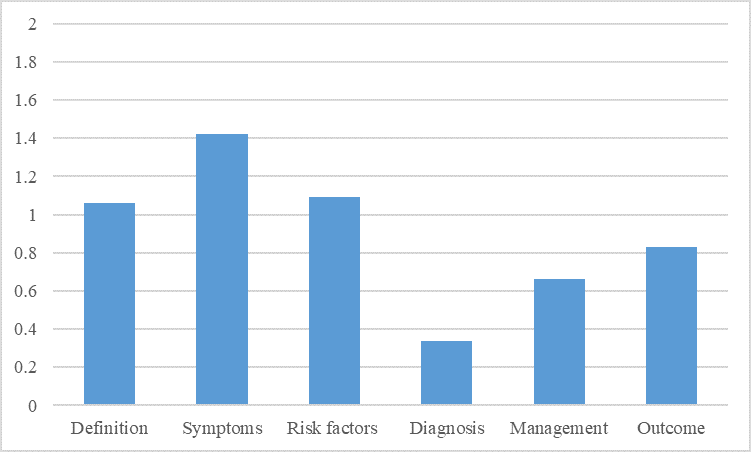

Supplement: Multimedia Appendix 2 [file jmir_v24i9e39360_app2.png]

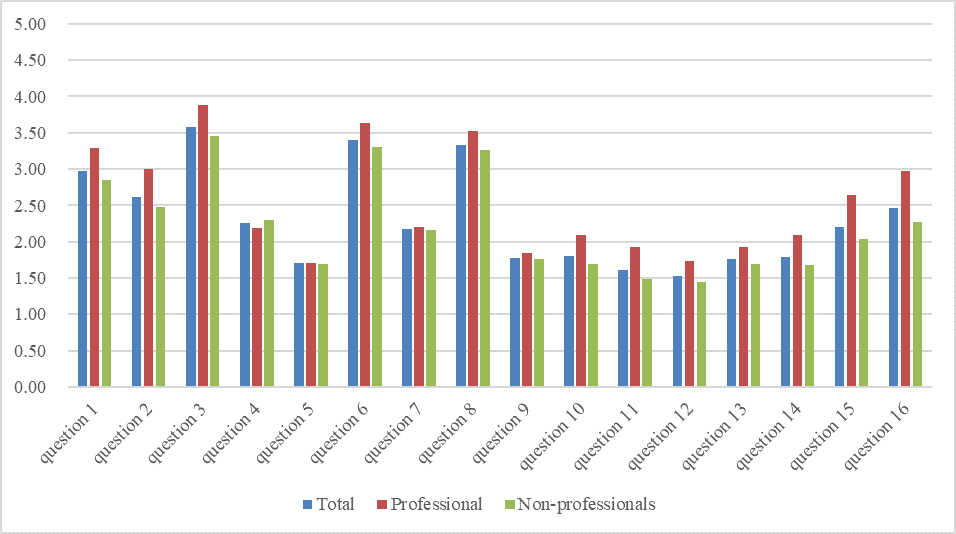

Supplement: Multimedia Appendix 3 [file jmir_v24i9e39360_app3.png]
